# HallmarkGraph: a cancer hallmark informed graph neural network for classifying hierarchical tumor subtypes

**DOI:** 10.1093/bioinformatics/btaf444

**Published:** 2025-08-13

**Authors:** Qingsong Zhang, Fei Liu, Xin Lai

**Affiliations:** School of Software Engineering, South China University of Technology, Guangzhou, 510006, China; School of Software Engineering, South China University of Technology, Guangzhou, 510006, China; Systems and Network Medicine Lab, Biomedicine Unit, Faculty of Medicine and Health Technology, Tampere University, Tampere, 33520, Finland; Department of Dermatology, Universitätklinikum Erlangen and Friedrich-Alexander-Universität Erlangen-Nürnberg, Erlangen, 91054, Germany

## Abstract

**Motivation:**

Accurate tumor subtype diagnosis is crucial for precision oncology, yet current methodologies face significant challenges. These include balancing model accuracy with interpretability and the high costs of generating multi-omics data in clinical settings. Moreover, there is a lack of validated models capable of classifying hierarchical tumor subtypes across a comprehensive pan-cancer cohort.

**Results:**

We present a graph neural network, HallmarkGraph, the first biologically informed model developed to classify hierarchical tumor subtypes in human cancer. Inspired by cancer hallmarks, the model’s architecture integrates transcriptome profiles and gene regulatory interactions to perform multi-label classification. We evaluate the model on a comprehensive pan-cancer cohort comprising 11 476 samples from 26 primary cancers with 405 subtypes up to eight levels. The model demonstrates exceptional performance, achieving 5-fold cross-validation accuracy between 85% and 99% for tumor subtypes labeled with increasing details of genomic information. It also shows good generalizability on a validation dataset of 887 samples, assessed using three metrics that consider tumor subtypes at individual, combined, and sample levels. Benchmarking and ablation experiments show that hallmark-based embeddings slightly influence model performance, while the integrated multilayer perceptron plays a significant role in determining classifier accuracy. Additionally, we use the SHAP method to link cancer hallmarks with genes, identifying key features that influence model decisions. Our findings present a biologically informed machine learning framework capable of tracking tumor transcriptomic trajectories and distinguishing inter- and intra-tumor heterogeneity in pan-cancer. This approach holds promise for enhancing cancer diagnostics.

**Availability and implementation:**

HallmarkGraph is accessible at https://github.com/laixn/HallmarkGraph.

## 1 Introduction

Cancer represents a complex spectrum of genetic diseases characterized by substantial phenotypic diversity and genetic heterogeneity, both across and within cancer types (Reiter *et al.* 2019, Zhao *et al.* 2019). While molecular subtyping has enabled the stratification of cancers into seemingly homogeneous groups with shared molecular and clinical characteristics, current diagnostic approaches lack robust classifiers that can accurately predict hierarchical tumor subtypes based on molecular features. The implications of imprecise tumor subtype diagnosis are significant, potentially leading to suboptimal treatment decisions and compromised patient outcomes. This underscores the critical need for enhanced tumor subtype diagnostic methodologies to support precision oncology decision-making (Mateo *et al.* 2022).

The development of predictive models for cancer subtype classification encompasses diverse methodological approaches (Zhao *et al.* 2019, Garofano *et al.* 2021, Lai *et al.* 2022, Shen *et al.* 2022, Li and Nabavi 2024). Contemporary research in cancer subtype classification primarily follows two distinct paradigms. One leverages advanced statistical and machine learning algorithms to classify tumor subtypes using single-modal data, such as gene expression profiles and histological images (Gogoshin and Rodin 2023, Wang *et al.* 2024, Xu *et al.* 2024). The other integrates multi-modal data, such as genome-wide transcriptome and epigenome, to utilize their complementarity to capture complex molecular interactions that define cancer subtypes (Lipkova *et al.* 2022, Stahlschmidt *et al.* 2022, Vera *et al.* 2022, Waqas *et al.* 2024,  Ellrott et al., 2025). For instance, Perou *et al.* first proposed a method for classifying four subtypes of breast cancer based on gene expression profiles (Perou *et al.* 2000). Recently, Jin *et al.* developed a convolutional neural network that integrates patients’ multi-omics and image data to classify HR+/HER2− breast cancers and identifies four subtypes with distinct biological and clinical features (Jin *et al.* 2023). The available models have demonstrated promising results in tumor subtype classification, but significant challenges persist. These include the inherent trade-off between model accuracy and interpretability, as well as the substantial costs associated with data generation, particularly for multi-omics profiling in clinical settings. Furthermore, although gene expression-based subtyping is proposed in individual cancer types (Zhao *et al.* 2019), a notable gap exists in the current literature is the absence of validated models capable of classifying hierarchical tumor subtypes across a comprehensive pan-cancer cohort.

Here, we present a graph neural network model, called HallmarkGraph, to classify tumor subtypes with hierarchy. The development of the model is inspired by cancer hallmarks, which are functional capabilities that are crucial for human cells to form malignant tumors (Hanahan 2022). This approach allows the model to integrate transcriptome profiles with gene regulatory interactions to build tumor classifiers. We test the model on a comprehensive pan-cancer cohort with >10 000 samples originating from 26 primary cancer types and containing 405 subtypes. The model shows excellent performance in classifying tumor subtypes, achieving a 5-fold cross-validation accuracy ranging from 85% to 99% for eight individual classifiers. The model also shows good generality on a validation dataset of 887 samples evaluated using three proposed metrics that account for tumor subtypes at the individual, combined, or sample levels. Finally, we characterize the contribution of cancer hallmarks with genes’ SHAP values, identify key genes that influence model decisions, and elaborate on the genes’ biomedical relevance. Taken together, our study introduces the first cancer hallmark-informed deep learning framework capable of hierarchically classifying pan-cancer tumor subtypes across up to eight levels with robust performance. By integrating cancer hallmarks into a graph neural network, HallmarkGraph not only achieves high accuracy but also provides biologically interpretable insights by tracing predictions from genes to hallmarks, significantly enhancing model interpretability and clinical relevance.

## 2 Materials and methods

### 2.1 Data collection and processing

We download the gene expression data from the Treehouse database (Comitani *et al.* 2023) that is an integrative cohort that collects and annotates patients from TCGA (ICGC/TCGA Pan-Cancer Analysis of Whole Genomes Consortium 2020) and TARGET. The data includes 12 637 samples, each having an expression profile of 44 793 genes, from a pan-cancer cohort comprising 26 cancer types. The gene expression is measured using the Kallisto method followed by log2 (TPM + 1) normalization.

The original sample labels are taken from the study (Comitani *et al.* 2023) and classified into different stages of tumors with morphological and genomic information based on a hierarchical labeling system. Basically, sample labels start with an identifier and abbreviated tumor sites of origin (e.g. T000 CNS for central nervous system carcinoma and T013 for melanoma), followed by subtype classification based on histologic classification and genetic mutation (e.g. T030 GLI IDHwt means glioma with IDH1 mutations). For the 26 tumors, we obtain their subtype labels varying from level 1 to level 8 (Table 1, available as supplementary data at *Bioinformatics* online). Level 1 means the primary tumor type and level 8 means the tumor contains subtypes up-to 8 tiers. Each tier is a subtype of its upper-level subtype and may contain several labels. For instance, Leukemia (LEUK; the primary level) samples have two major subtypes (i.e. level 2)—ALL and AML. The two subtypes, depending on morphological phenotypes and gene mutations, have up to 8 and 4 levels, respectively (Excel 1, available as supplementary data at *Bioinformatics* online).

**Table 1.** Performance of the classifiers on the test and validation datasets.^a^

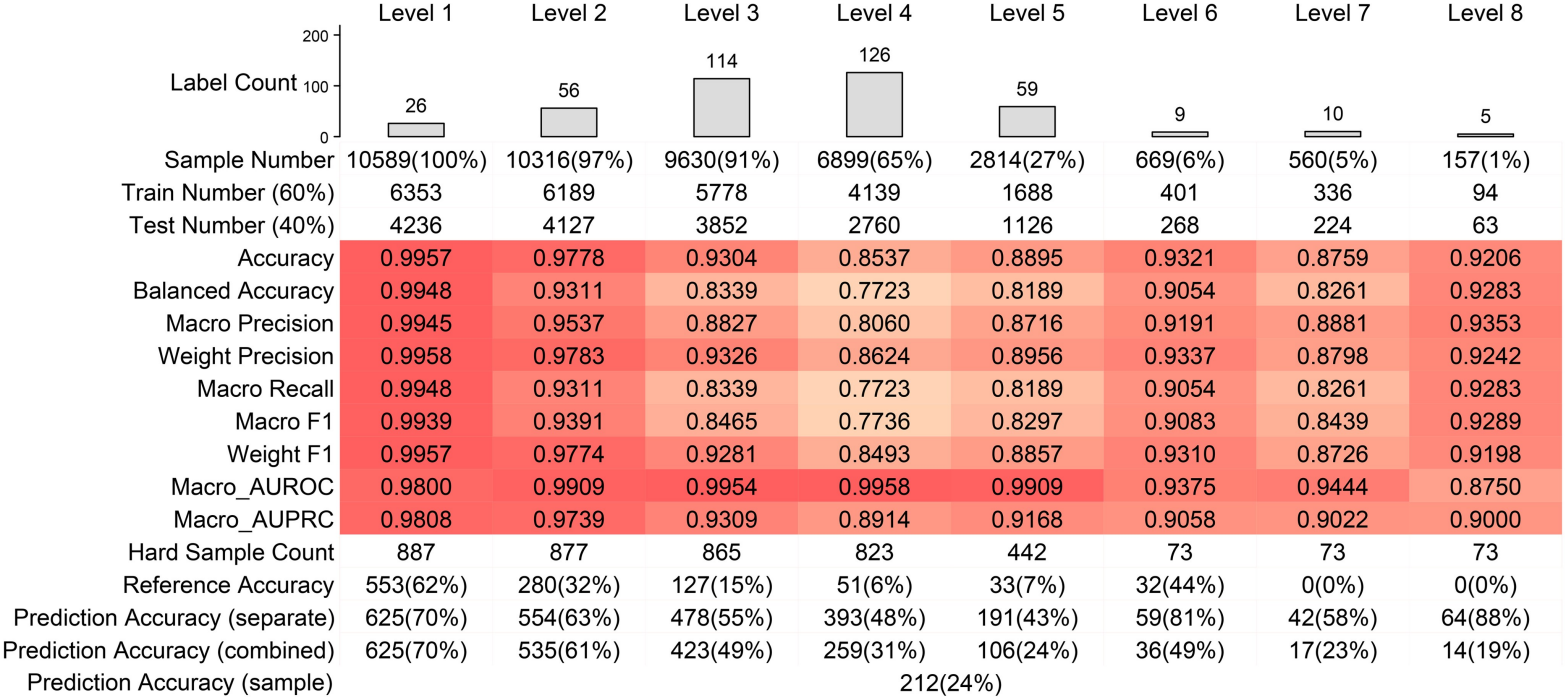

a The table presents a comprehensive statistical breakdown of label and sample distributions across classifiers, revealing a progressive reduction in samples as label complexity increases from level 1 to level 8. It also includes an average of nine metrics derived from the 5-fold cross-validation test datasets, with color intensity denoting metric values. The complete metrics can be found in Excel 4, available as supplementary data at *Bioinformatics* online and the corresponding AUROC and AUPRC curves for each label level are shown in Fig. 4, available as supplementary data at *Bioinformatics* online, respectively. The statistics of validation dataset include sample distribution, reference accuracy from the original study, and the classifiers’ metrics at individual, combine, and sample levels. The classification on 887 individual samples and statistics on different cancer types can be found in Excel 5a and b, available as supplementary data at *Bioinformatics* online, respectively.

As a result, we obtain different numbers of samples for labels at each level (Table 2, available as supplementary data at *Bioinformatics* online), ranging from >10 000 for the primary classification (i.e. level 1) to a few hundred for the finest classification (i.e. level 8). While progressing sample labels from the literature, we identify nine samples with errors, typos or duplicates, and we therefore correct them manually (Table 3, available as supplementary data at *Bioinformatics* online). We also exclude samples that have the same original diagnosis and fewer than three, because the small sample number can make the classifiers unreliable. As a result, we obtain 11 476 valid samples (Excel 2, available as supplementary data at *Bioinformatics* online), and each has a transcriptomic profile of 6448 genes after filtering with cancer hallmark gene sets (see Section 2.2). We use UMAP to cluster and visualize the patients’ transcriptome profiles (McInnes *et al.* 2020).

**Table 2.** Benchmarking and ablation experiments.^a^

		Benchmarking			Ablation		
Level	Sample	HallmarkGraph	MLP	CNN	HallmarkGraph-3	HallmarkGraph-2	HallmarkGraph-1
Level 1	10 589	0.9957±0.0028	**0.9968±0.0002**	0.9964±0.0008	**0.9957±0.0028**	0.9894±0.0036	0.9944±0.0021
Level 2	10 316	0.9778±0.0014	**0.9782±0.0012**	0.9768±0.0015	**0.9778±0.0014**	0.9738±0.0048	0.9683±0.0063
Level 3	9630	**0.9304±0.0102**	0.9125±0.0359	0.9288±0.0062	**0.9304±0.0102**	0.9232±0.0041	0.9095±0.0103
Level 4	6899	**0.8537±0.0445**	0.8524±0.0210	0.8402±0.0431	**0.8537±0.0445**	0.8389±0.0066	0.8385±0.0117
Level 5	2814	0.8895±0.0037	**0.902±0.01120**	0.8826±0.0035	**0.8895±0.0037**	0.8426±0.0286	0.8599±0.0177
Level 6	669	0.9321±0.0097	**0.9634±0.0017**	0.9194±0.0034	0.9321±0.0097	0.9456±0.0050	**0.9463±0.0034**
Level 7	560	0.8759±0.0020	**0.8768±0.0040**	0.8589±0.0040	0.8759±0.0020	0.8768±0.0360	**0.8911±0.0060**
Level 8	157	**0.9206±0.0159**	0.8413±0.0355	0.8254±0.0000	**0.9206±0.0159**	0.8508±0.0142	0.8667±0.0142

a The table shows accuracy values (mean ± SD) of the classifiers’ performance on the test dataset after training the model with the 5-fold cross-validation approach for 5 times. The bold fonts correspond to the best performing model per row. For benchmark tests, all models have a 3-layer MLP. To make the convolution neuron network (CNN) embedding comparable with HallmarkGraph, we use 10 convolutional kernels (as HallmarkGraph has 10 hallmarks), set the convolutional kernel size 1×1 (consistent with HallmarkGraph’s network weights that are identical and shared), and set padding to ‘same’ to maintain the original number of features after information convolution. The rest of the hyper-parameters are set the same as HallmarkGraph. For ablation experiments, the numerical suffix in each model’s name denotes the number of MLP layers. Specifically, HallmarkGraph-3, -2, and -1 correspond to MLP architectures with the following structures: (i) 512 → 256 → [number of labels], (ii) 256 → [number of labels], and (iii) [number of labels], respectively. Except for the accuracy values, the other evaluation metrics can be found in Excel 5c, available as supplementary data at *Bioinformatics* online.

**Figure 1.**
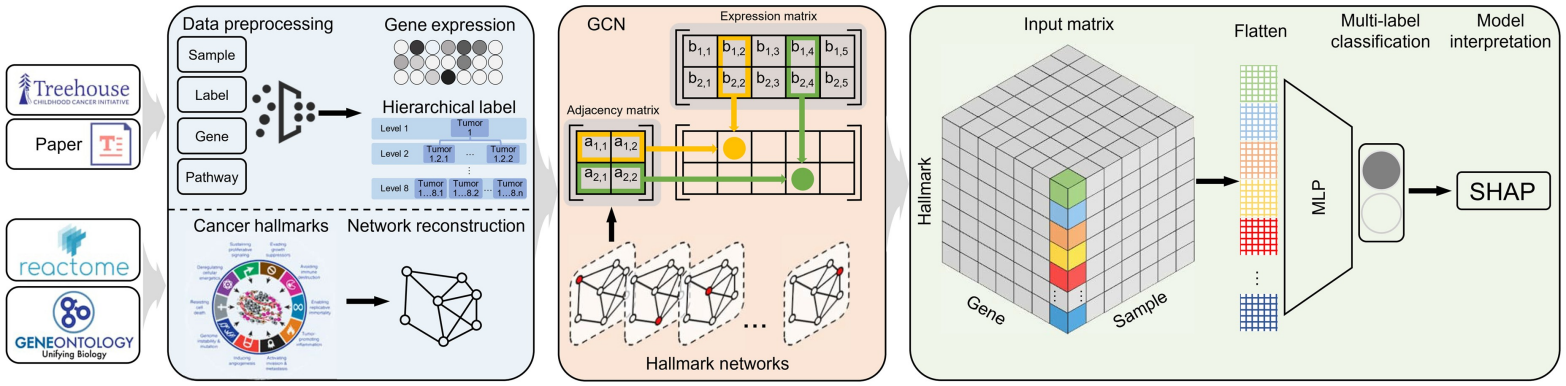
Overview of the computational pipeline. First, we process the cohort data, including label cleaning, sample grouping, and reconstruction of cancer hallmark networks. Second, we use a graph convolution network (GCN), which integrates the expression levels of genes and their interacting partners in the hallmark networks to embed feature values. We then train the model to classify labels at eight different levels, resulting in eight classifiers, and evaluate their performance. Finally, we use SHAP to quantify the contribution of genes and their associated cancer hallmarks in the models.

We then categorize these valid samples into two groups: TRUE and FALSE, based on the correspondence between their labels and the original diagnosis from the study (Comitani *et al.* 2023). To achieve this, we first subdivide the hierarchical labels of the samples into the respective levels (i.e. 1–8). Second, we undertake a search for characters that align with clinical diagnosis across different tier labels. If the characters appear in all labels, the sample is categorized as a TRUE sample; otherwise, it is categorized as a FALSE sample. For instance, an SKCM sample has four labels from level 1 to 4. The sample is categorized as a TRUE sample if its level 1 label is MELA and level 2–4 labels contain SKCM, or as a FALSE sample if any corresponding label does not match MELA or SKCM (Fig. 1, available as supplementary data at *Bioinformatics* online). Third, the FALSE samples’ primary labels are corrected to match their clinical diagnosis, and their remaining tier labels are removed. This ensures that all FALSE samples are correctly annotated at the primary level, but their subtype classifications are ignored. However, there are some exceptions to this rule. If a FALSE sample has more than one label in level 1 that is assigned in the original article, its labels are not reliable and then are deleted. Only its diagnosis label is considered reliable and used for model validation. Finally, the TURE samples are utilized for model training, while the FALSE samples, which are considered challenging due to their erroneous classification in the original paper, are used for model validation and comparison. It is noteworthy that a label (i.e. T080 SARC IMMhigh) is identified with only one sample, it is not suitable for training classifiers and is therefore classified as a FALSE sample.

### 2.2 Cancer hallmark gene sets and network construction

**Figure 2.**
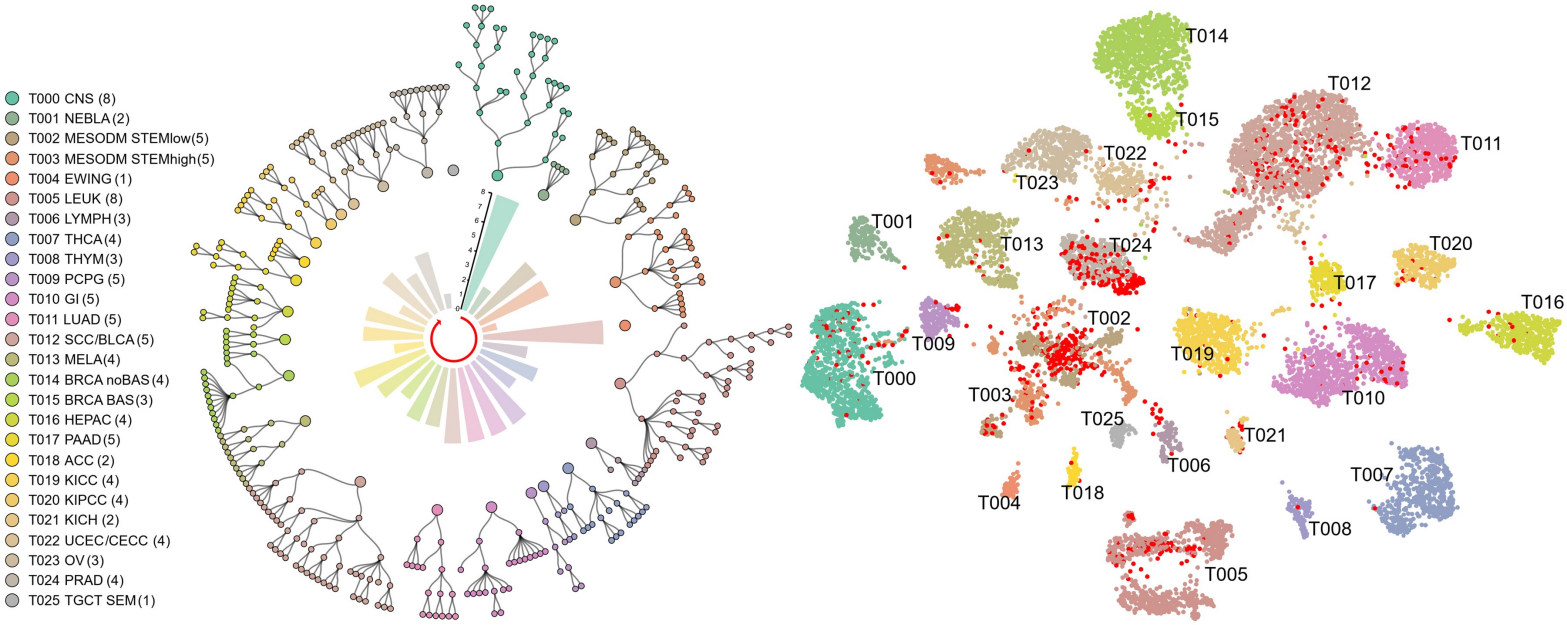
Overview of sample labels. The tree diagram shows hierarchical labels of tumor subtypes, with the depth of the tree corresponding to the tier level. Each tree depicts a specific tumor type, with a left-aligned legend indicating the maximum tier of sample labels by number. The central bar plot quantifies the tree diagram’s depth structure. A red arrowed line at the center indicates the reading order from T00 CNS to T025 TGCT SEM. The right UMAP plot illustrates the clustering of 11 476 tumor samples, with 887 FALSE samples (red dots). The tumors’ identifiers in both plots are the same and their full names can be found in Table 1, available as supplementary data at *Bioinformatics* online.

We use GO terms representing ten cancer hallmarks to construct gene sets (Chen *et al.* 2021). We use official gene names for each cancer hallmark gene set from the Ensemble database (GRCh38.p14; v112) (Harrison *et al.* 2024). We identify 6448 genes that are in the curated gene sets and have expression values derived from the Treehouse database. As a result, we get genes that are shared by and specific to cancer hallmarks (Fig. 2, available as supplementary data at *Bioinformatics* online). To construct hallmark-specific networks, we use the Reactome database to download functional interactions among genes (v061424) (Milacic *et al.* 2024). We filter the interactions using the 6448 genes in cancer hallmarks, resulting in 75 869 directed molecular interactions. The resulting networks are graphs that are used to compute message-passing among genes for each hallmark (Table 4, available as supplementary data at *Bioinformatics* online). This information, along with gene expression profiles, is used to develop HallmarkGraph with a graph neural network architecture.

### 2.3 HallmarkGraph construction, training, evaluation, and validation

For each hallmark network, we use a graph convolution method to compute the message-passing between genes, considering the network as undirected. Specifically, for each gene, we sum the expression of its direct neighbors and use it as the gene’s feature value [Equation (1)]. If a gene has no neighbors (i.e. interactions) in a cancer hallmark network, we use the gene’s own expression value as its feature value. This process is called residual connections (He *et al.* 2016).

(1) $$\begin{array}{c} f(H^{\left(l\right)},{\bar{A}}_{\mathrm{hallmark}})=\\ \sigma ({\bar{A}}_{\mathrm{hallmark}}H^{\left(l\right)}W^{\left(l\right)})=\\ R\mathrm{elu}((I_{n}+D^{-\frac{1}{2}}A_{\mathrm{hallmark}}D^{-\frac{1}{2}})H^{\left(l\right)}W^{\left(l\right)}) \end{array}$$

where *A_hallmark_* is the adjacency matrix of a hallmark network. *H^(l)^* and *W^(l)^* are the input matrix and weight matrix of the *l*th aggregation, respectively. *I_n_* is the unit matrix and *D* is the degree matrix.

After that, we obtain ten matrices with the same dimension (i.e. 6448 x sample) for each cancer hallmark. The difference between the matrices reflects the genomic and topological specificity of the hallmark networks, which can lead to different values in genes. We extend the matrices by aggregating samples’ data, resulting in a 3D matrix containing 6448 genes, 10 cancer hallmarks, and samples that vary in size for different levels of tumor subtypes. The 3D matrix is connected to a multilayer perceptron (MLP) with three fully connected layers, where the first two layers have 512 and 256 neurons, respectively, and the last layer has neurons equal to the number of labels predicted by the classifier. The last layer of the neural network is connected to a softmax function to predict the probability of labels used to train the model. The label predicted with the highest probability is assigned to a sample.

**Figure 3.**
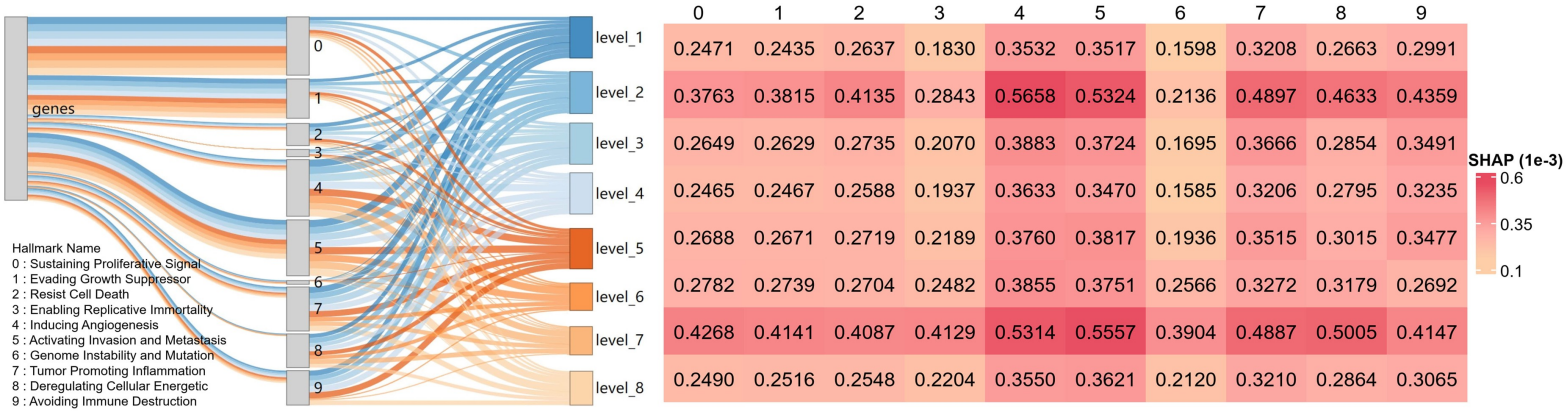
SHAP analysis at the hallmark level. The Sankey plot visualizes gene contributions across hallmarks and their aggregated impact on eight classifiers, with normalized gene contributions based on averaged absolute SHAP values. Each classifier’s total SHAP values equal to 1, and hallmark contributions are calculated by dividing summed SHAP values by gene count, followed by min-max normalization. Line width represents the relative contribution of genes or hallmarks to each classifier, with color-matched lines enabling direct comparisons within classifiers. The table provides detailed aggregated SHAP values for each hallmark (column) across classifiers (row).

For labels in each tier-level, we train a classifier using the samples from the TRUE group (Table 2, available as supplementary data at *Bioinformatics* online). The resulting eight multi-label classifiers with the same architecture can predict samples’ tumor subtypes at the corresponding level. We use Adam optimizer, a stochastic optimization method (Kingma and Ba 2017), to obtain the optimal values of hyperparameters including learning rate = 1e−3 and batch size = 200. We fix the number of epochs at 200. To avoid overfitting, we adopt early stop that the model training terminates when the cost function’s value does not decline in five consecutive epochs. During the training, we split the data into 60% training and 40% test datasets by ensuring the proportion of labels from different cancer types are consistent with the original dataset (Fig. 3, available as supplementary data at *Bioinformatics* online). We use 5-fold cross-validation to evaluate the model’s performance using the test data. The models are trained to minimize the categorical cross-entropy loss function $L(y,\hat{y})=-\sum_{i=1}^{C}y_{i}\log({\hat{y}}_{i})$, where y is the one-hot coding true label, $\hat{y}$ is the model prediction, and *C* represents the total number of labels of the corresponding tier level. We train each classifier 5 times on a workstation with two 12 GB NVIDIA 2080Ti GPUs and the total training time is about one hour. From the 5-fold cross-validation, we consider the model with the maximum value of balanced accuracy as the best model. We use the best model on the test data to obtain confusion matrices and draw receiver operating characteristic (ROC) and precision-recall (PRC) curves. We also systematically evaluate the models’ performance using metrics such as accuracy, balanced accuracy, weighted F1, micro and macro precision, recall, F1, AUROC, and AUPRC (Table 5, available as supplementary data at *Bioinformatics* online).

Finally, we use the best performing model to predict the 887 samples from the FALSE group that are excluded for model training and considered as a validation dataset. We apply this rule to three metrics, namely separate, combined and sample-level accuracy, which can validate the model’s performance with or without considering sample labels’ hierarchal relationships (see Supplementary Materials for details; Excel 3, available as supplementary data at *Bioinformatics* online).

### 2.4 Feature ranking and model intepretation

We use SHapley Additive exPlanations (SHAP) to characterize the importance of genes to our classifiers (Lundberg and Lee 2017). The algorithm uses Shapley scores derived from game theory to quantify the contribution of features to a model. We compute the SHAP values (with default parameter values) for features in all classifiers trained on the samples in the TRUE group and rank them based on their absolute SHAP values—the higher values a gene has, the more important it is to a classifier. Since our features are genes in cancer hallmarks, we can further associate them and quantify the importance of the hallmarks for each tier level label. To do so, we first sum the absolute SHAP values of genes for each hallmark. We then calculate the average of the summed SHAP values by dividing them with the number of genes in each hallmark. This results in the hallmarks’ contribution to the classifiers.

### 2.5 Data visualization

We develop our model using Tensorflow (v2.8) in Python (v3.11). For data visualization, we use R package ComplexHeatmap (Gu *et al.* 2016), ggplot2 (Wickham 2016), and networkD3 (Allaire *et al.* 2017).

## 3 Results

We develop a computational pipeline that focuses on classifying tumor samples of a pan-cancer cohort (Fig. 1). The labels are biologically relevant, reflecting the hierarchical classification of tumor subtypes based on cancer patients’ molecular profiles. The pipeline includes three modules: (i) data cleaning and processing, (ii) classifier construction, and (iii) classifier training, prediction, and interpretation.

### 3.1 Hierarchical tumor subtypes in a pan-cancer cohort

Comitani and co-authors develop a scale-adaptive clustering approach to identify tumor subtypes based on transcriptomic data, resulting in a pan-cancer cohort of sorted subtypes (Comitani *et al.* 2023). The comprehensive data allows us for developing classifiers to differentiate hierarchical tumor subtypes. Toward this goal, we clean up the original data by removing all normal samples and tumor types with too few samples. We further correct a few mislabeling samples with duplicating names and mismatching information between mother and child labels (see Section 2.1). As a result, we obtain a dataset of 26 primary tumors of 11 476 samples, and each tumor sample has a hierarchical label containing up to 8 tier levels. They are further divided into TRUE or FALSE groups, where the TRUE samples are the training dataset, and the FALSE samples are the validation dataset (see Section 2.1). For most cancers, their labels have less than five levels, two cancers (i.e. EWING and TGCT SEM) have only primary labels, and two cancers (i.e. CNS and LEUK) have 8 tier levels (Fig. 2). Samples from the same tumor origin cluster together due to similar transcriptomic profiles and differ from samples from other cancer types. This suggests that differentiating subtypes of the same primary tumor is a challenging task. In addition, we identify 887 samples that are misclustered in the original study, primarily from mesodermal (MESODM), leukemia (LEUK), bladder (BLCA) and pancreas (PRAD) cancer patients.

### 3.2 HallmarkGraph shows prominent ability to differentiate tumor subtypes

The pan-cancer patient cohort reveals intricate hierarchical tumor subtype variations, presenting a nuanced classification challenge characterized by complex intra- and inter-tumoral heterogeneity. Therefore, we aim to develop classifiers that can distinguish the tumor hierarchy of the cohort using patients’ transcriptomic profiles. Briefly, we first annotate gene sets for cancer hallmarks that represent the known and shared phenotypic properties of tumors. Second, we reconstruct hallmark networks using molecular interactions from the Reactome database. The interactions are derived from expert-curated pathways, representing consensus knowledge of biological processes and signaling pathways in cells. Third, we apply a GCN for feature embedding that integrates the expression of hallmark networks’ genes and their interacting partners. The embedding features are connected to a fully connect neural network to form a multi-label classifier. The classifier, called HallmarkGraph, is a biologically informed neural network because its framework is constrained by the topological properties of hallmark networks. Finally, we train the classifier for each tumor subtype level using 60% of TRUE group samples, evaluating performance on the remaining 40% TRUE samples and testing predictive capability with a validation dataset of 887 FALSE group samples (see Section 2.3 for details).

The resulting eight classifiers show exceptional performance in classifying tumor subtypes at different levels, achieving remarkably high accuracy metrics (Table 1). As the number of labels increases from level 1 to level 4, a corresponding moderate decline in performance metrics emerges, reflecting the inherent challenges of progressively more nuanced tumor subtype classification tasks. Despite this reduction in metrics such as macro-precision, macro-recall, and F1 scores, the classifiers maintain robust performance, as indicated by consistently high values of macro AUROC. When the label level goes up from 5 to 8, the performance of the models increases again due to the significantly reduced number of labels. Although they are not as good as the classifiers for levels from 1 to 3, they still maintain an excellent performance reflected by all metrics. The differing performance could be due to reduced sample sizes in those higher-level labels that provide more detailed tumor characteristics. It could also be affected by reduced labels, but to a much lesser extent compared to the reduction in sample sizes. More specifically, the classifiers have the maximum and minimum accuracy of 0.9957 and 0.8537, respectively. The best performing classifier is for the primary labels (i.e. level 1), which is like a pan-cancer classifier to differentiate 26 TCGA cancer types and shows an accuracy of 0.9957, which is superior to other studies (Mostavi *et al.* 2020, Ramirez *et al.* 2020, Divate *et al.* 2022). The classifier for the level 4 labels has the lowest accuracy of 0.8537 but a macro AUROC of 0.9958. This means the classifier can effectively distinguish between positive and negative tumor subtypes (i.e. predicting high probability of the positive labels for each binary classification of the 126 labels) but its overall accuracy is being impacted by the multi-label complexity and potential imbalance in label prevalence. Similar results are also observed in the classifier for level 5 labels which has an accuracy of 0.8537 and a macro AUROC of 0.9909. Furthermore, we test the classifiers using a validation dataset (i.e. the 887 FALSE group samples). We use three metrics to evaluate the performance of the classifiers, demonstrating the model’s ability to classify tumor subtypes at individual, combined, and sample levels (see Section 2.3 for details). The classifiers show a significant performance improvement over the original study on the FALSE samples at all levels (Table 1). When predicting tumor subtypes at the individual tumor subtype level, our models are generally more accurate (ranging from 8% to 88%) than the original study. The level 7 and 8 classifiers show the best performance, achieving an 88% prediction accuracy on 73 FALSE samples, in stark contrast to the original study’s completely unsuccessful labeling (0% accuracy). The smallest improvement occurs with the level 1 classifier for 887 samples, where the accuracy increases by 8% from 62% to 70%. When the tumor subtypes are predicted in a combined manner (i.e. by linking the labels at different levels), the performance of the models becomes slightly worse. The increase in prediction accuracy drops to between 5% and 29%. The least and most significant increase in prediction accuracy occurs in the level 6 and level 2 classifiers, and they increase from 44% to 49% and from 32% to 61%, respectively. Finally, using sample-level accuracy for evaluation, the models correctly predict the labels of 224 samples, or 24% of the 887 FALSE samples. Overall, the models demonstrate robust generalizability using the validation dataset despite some limitations in predicting hierarchical labels.

Taken together, HallmarkGraph shows the robust performance of across all hierarchical levels of tumor subtype classification, demonstrated by high accuracy of the classifiers that can differentiate tumor subtypes with different genomic details. Although the sample size for different classifiers imbalances (e.g. the level 8 classifier has only 157 samples compared to >10 000 samples of the level 1 classifier), the cross-validation with 60% training and 40% test and significantly improved performance on the validation dataset (i.e. the 887 samples) prove the reliability of the model and mitigate its potential overfitting for nuanced tumor subtypes.

### 3.3 Model interpretation from genes to cancer hallmarks

HallmarkGraph uses a GCN that leverages the topological characteristics of hallmark networks for feature embedding, thereby integrating intrinsic biological network properties. This approach enables a sophisticated analysis of model decisions by combining advanced machine learning techniques with established biological domain knowledge. For each classifier, we first use the SHAP method to compute genes’ SHAP values and quantify their aggregated contribution in each hallmark (see Section 2.4 for details). The data show that the hallmarks 4 and 5 are the most important overall across all classifiers, and the hallmarks 3 and 6 are the least important (Fig. 3). This suggests that genes associated with angiogenesis and tumor invasion and metastasis contribute more to the hierarchical classification of tumor subtypes than other cancer hallmarks. In addition, the level 2 and 7 classifiers appear to rely more heavily on all ten hallmarks, although they do not have the largest number of labels. This suggests that the tumor subtypes from both levels may have high inter- and intra-transcriptomic complexity that require a combination of hallmarks to distinguish them. In contrast, the other classifiers show smaller hallmark contributions, suggesting more pronounced inherent distinctions within the transcriptomic profiles of their respective tumor subtypes.

Moreover, to gain insights at the gene level, we rank the genes of each classifier based on their absolute average SHAP values and analyze their overlap in all classifiers (Fig. 5, available as supplementary data at *Bioinformatics* online). The higher rank of a gene in a classifier the more important its transcriptomic expression contributes to classification of tumor subtypes at the same level. The overlap of the top-ranked 2000 genes is about 30% in all classifiers. This suggests that the classifiers choose different genes to differentiate tumor subtypes. If we divide them into two groups, the level 1–4 classifiers show a significantly higher intersection of genes than the level 5–8 classifiers. This is consistent with the expectation that the more nuanced tumor subtypes require more transcriptomic information to differentiate them.

**Figure 4.**
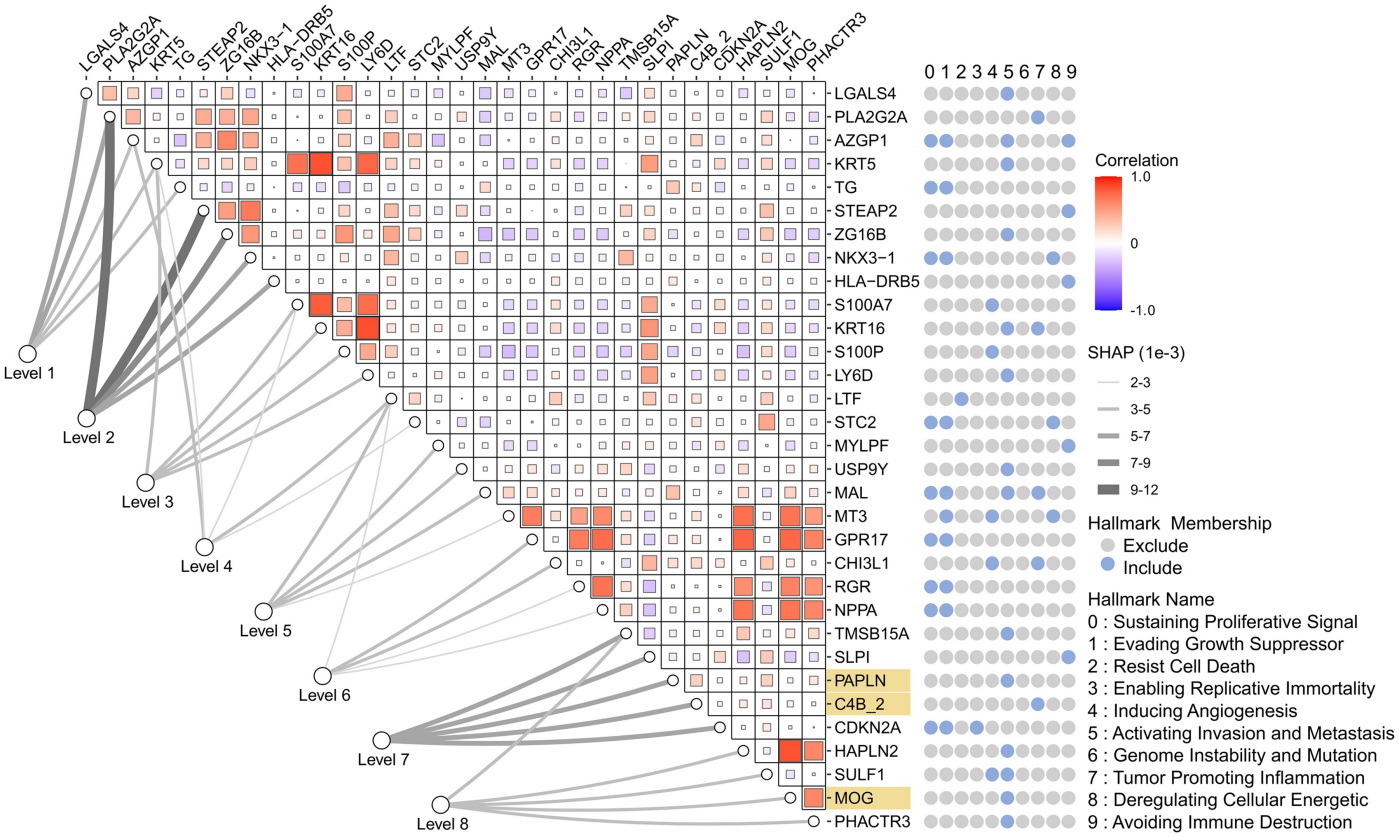
SHAP analysis at the gene level. The heatmap shows the Pearson correlation coefficient (represented by grid color and size) of the top-ranked five genes (empty circles) from each classifier (represented by lines whose width are quantified by SHAP values). The blue circles on the right side represent the association between genes and cancer hallmarks. The highlighted genes are not identified as tumor biomarkers from the literature search and the other genes are potential tumor biomarkers (Excel 6, available as supplementary data at *Bioinformatics* online).

We further analyze the top-ranked genes for each classifier and identify 32 genes from cancer hallmarks (Fig. 4). Of these, 19 genes are uniquely associated with a single hallmark, while 13 genes span multiple hallmarks. This distribution reveals that over half of the genes exhibit hallmark-specific characteristics, with the remaining genes demonstrating broader involvement across several hallmarks. For instance, AZGP1 is known to influence several cancer hallmarks including regulation of tumor proliferation (Tian *et al.* 2017), epithelial-mesenchymal transition (Deng *et al.* 2023), and infiltration of immune cells in tumor microenvironment (Hanamura *et al.* 2024). Most of the top-ranked genes show altered expression in tumor tissues and correlate with key clinicopathological features (Excel 6, available as supplementary data at *Bioinformatics* online). For example, upregulation of genes (e.g. CHI3L1, S100A7, and MAL) is associated with poor prognosis, increases aggressiveness, and tumor microenvironment modulation. Conversely, downregulation of genes, like LGALS4 and LTF, is linked to tumor progression and metastasis.

In addition, our analysis reveals that nearly all cancer hallmarks are represented by at least one gene, with hallmarks 5, 1, and 0 containing the most genes (15, 10, and 9, respectively), reflecting their broader gene composition. Most genes demonstrate high classifier specificity, indicating the need for distinct biomarkers when characterizing tumor subtypes at various levels of detail. Six genes are present in up to three classifiers: KRT5 and LTF appear in three classifiers, while PLA2G2A, AZGP1, S100A7, and TMSB15A are present in two. This distribution suggests these genes’ different roles in differentiating tumor subtypes. Notably, KRT5's presence in the level 1, 2, and 4 classifiers suggests its importance in coarse classification with limited genomic information, whereas LTF's involvement in the level 4, 5, and 6 classifiers indicates its significance in more refined, genomically comprehensive classifications.

The top-ranked genes across different classifiers exhibit distinct SHAP value distributions, with the level 2 classifier particularly notable for its larger variance in gene contributions. This characteristic is evident in the more dispersed SHAP value spread, contrasting with other classifiers’ more concentrated distributions (Fig. 6, available as supplementary data at *Bioinformatics* online). The level 2 classifier’s unique pattern stems from extreme gene contributions in the corresponding samples (Fig. 7, available as supplementary data at *Bioinformatics* online), suggesting a more pronounced dominance of top-ranked genes compared to other classifiers where gene contributions appear more uniformly distributed. Furthermore, certain classifiers reveal high gene correlations, indicating potential redundancy in genetic information. Specifically, the level 3 classifier shows strong correlations among genes like KRT5, S100A7, KRT16, S100P, and LY6D, while the level 6 classifier exhibits similar patterns with GPR17, RGR, and NPPA, highlighting the complex interplay of genes in tumor subtype classification.

Taken together, our analyses demonstrate that distinct gene signatures are instrumental in classifying hierarchical tumor subtypes. This finding suggests that differential gene expression patterns, manifested through transcriptomic profiles within cancer hallmarks, reflect the genomic evolutionary trajectories of tumors, enabling the discrimination of both inter- and intra-tumor heterogeneity.

### 3.4 Model benchmarking and ablation experiments

Previous research in cancer subtyping has predominantly focused on multi-label prediction models for individual cancer types, often using MLPs or GCNs without explicitly incorporating cancer hallmark information (Zhao *et al.* 2019). To benchmark our approach, we conduct comparative analyses of classification performance across these established models using eight distinct tumor subtype levels. As shown in Table 2, the classification accuracy of these models shows similar performance with minimal differences (<1%) for most tumor levels (i.e. tier levels 1–5 and 7). However, increased differences (∼3%–7%) are observed for specific tumor levels (i.e. tier levels 6 and 8). This suggests that in subtypes with abundant samples, the models exhibit robust and comparable performances, regardless of the inclusion of graph-based embeddings. However, when the number of samples significantly decreases, the models’ performances exhibit higher variance. In these low-sample conditions, HallmarkGraph demonstrates the best performance on tier level 8. Nonetheless, it is important to acknowledge that the generalizability and robustness of models, even those incorporating graph embeddings, may be compromised due to the inherently small sample sizes for more nuanced tumor subtypes.

To elucidate the contribution of individual components to the overall model performance, we conducted a series of ablation experiments. As detailed in Table 2, the exclusion of hallmark-based graph embeddings resulted in a noticeable performance reduction across 4 out of 8 classifiers. Furthermore, progressively decreasing the number of layers in the MLP within HallmarkGraph led to a consistent decline in model performance for 6 out of 8 classifiers. These findings underscore the critical role of both hallmark-based graph embeddings and the depth of the MLP architecture in determining HallmarkGraph’s efficacy.

Taken together, integrating prior domain knowledge into our model shows a limited impact on classifier performance. This might be due to redundant information within the cancer hallmark networks, suggesting a need for better distinction between true biological signals and noise. On the other hand, the depth of the MLP architecture proves crucial for model performance. Despite the modest impact of direct knowledge integration on overall classification, a key advantage of HallmarkGraph lies in its ability to enrich model interpretability, moving from high-level cancer hallmarks down to the contributions of individual genes.

## 4 Conclusions and discussion

In this work, we advance cancer classification through an approach that integrates prior biological knowledge with machine learning methods. HallmarkGraph is a graph neural network that considers cancer hallmarks into feature embedding, creating a robust method for classifying hierarchical tumor subtypes beyond current approaches. By differentiating complex tumor subtypes within a pan-cancer cohort, we distinguish ourselves from existing models that typically address non-hierarchical classifications (Li *et al.* 2017, Zhao *et al.* 2020). Despite the model’s impressive performance on a large pan-cancer cohort encompassing over 10 000 patients and 405 tumor subtypes, our study is constrained by a limitation—the absence of an independent dataset with comparable tumor hierarchy for validation. This constraint compromises the comprehensive assessment of the model’s generalizability. However, we may use methods to synthesize an independent validation cohort, which is not the current focus of the study (Bej *et al.* 2021). Future model development should prioritize chain-label prediction frameworks that transcend individual classifier limitations (Wehrmann *et al.* 2018). Such an approach would capture the inherent sequential and interdependent characteristics of hierarchical tumor subtypes, potentially enhancing predictive accuracy and offering a more holistic computational strategy for tumor classification. The integration of genome-wide transcriptome and gene regulatory interaction in HallmarkGraph enables unprecedented model interpretation across genetic and cancer hallmark dimensions. In conclusion, HallmarkGraph’s ability to stratify intricate tumor subtypes while maintaining interpretability represents a significant methodological advancement, holding promise for refining cancer diagnosis.

## Supplementary Material

btaf444_Supplementary_Data

## Data Availability

Hallmark is accessible at https://github.com/laixn/HallmarkGraph. The data and model are archived at https://doi.org/10.5281/zenodo.15790122.
