# Supplementary material for: HallmarkGraph: a cancer hallmark informed graph neural network for classifying hierarchical tumor subtypes
Source: Bioinformatics. 2025 Aug 13;41(9):btaf444. doi: 10.1093/bioinformatics/btaf444 (PMC12401579; doi:10.1093/bioinformatics/btaf444)
Supplement: btaf444_Supplementary_Data [file btaf444_supplementary_data.zip › 04a_hallmarkgraph_SM-text_r1.docx]

**Supplementary Materials**

**HallmarkGraph: a cancer hallmark informed graph neural network for classifying hierarchical tumor subtypes**

Qingsong Zhang^1^, Fei Liu^1,*^, Xin Lai^2,3,*^

^1^School of Software Engineering, South China University of Technology, Guangzhou, China

^2^Systems and Network Medicine Lab, Biomedicine Unit, Faculty of Medicine and Health Technology, Tampere University, Tampere, Finland

^3^Friedrich-Alexander-Universität Erlangen-Nürnberg and Universitätklinikum Erlangen, Erlangen, Germany

**^*^Corresponding author:** South China University of Technology, Guangzhou, China [feiliu@scut.edu.cn](mailto:feiliu@scut.edu.cn) (FL). Faculty of Medicine and Health Technology, Tampere University, Tampere, Finland. Email: and [xin.lai@tuni.fi](mailto:xin.lai@tuni.fi) (XL).

[**Supplementary text** 2](#_Toc193959162)

[**Supplementary tables** 5](#_Toc193959163)

[**Supplementary figures** 10](#_Toc193959164)

## **Supplementary text**

**Model validation using the FALSE group samples**

We perform model validation using the 887 samples in the FALSE group. We sort out tumor hierarchical levels by arranging them according to available clinical diagnosis of samples, this results in a reference table that is used to determine whether the classifiers’ predictions on FALSE samples are correct (SM Excel S3). Specifically, the rows in a color section represents a hierarchical tree of a particular tumor and its subtypes. Each row of the color section represents a tumor branch that is used to determine whether a FALSE is correctly predicted (see the following paragraph for details). A primary tumor (level 1) may contain several major subtypes (level 2), for example, LEUK has two major subtypes, ALL and AML. The major subtype AML can be further divided into two branches (level 3) including AMKL and other nine subtypes directly named after AML. The former branch has a maximum level of 4 and the latter has a maximum level of 5. As a result, we get the maximum hierarchical level of each tumor (see the ‘right max level’ column in SM Excel S3). Additionally, for some tumor branches we provide specific explanation on how a FALSE sample is handled when it is classified into one of the tumor branches (see the ‘explanation’ column in SM Excel S3).

All samples have eight prediction labels from the eight classifiers. A sample’s labels are considered valid for model evaluation only if they are equal to and less than its maximum level *n*, as determined by the clinical diagnosis of the sample (see the ‘right max level’ column in SM Excel S3). For example, if a sample has a clinical diagnosis of READ, its corresponding maximum prediction level is 3, so its prediction labels up to level 3 are used for model evaluation and its higher-level labels (i.e., 4-8) are discarded (Figure S8). We apply this rule to three metrics, namely separate, combined and sample-level accuracy.

For **separate accuracy**, if a sample has a clinical diagnosis of AMKL, its maximum tumor subtype level is 4, so we independently evaluate its prediction labels from level 1 to 4 classifiers. Its prediction labels by the level 1, 2, and 3 classifiers are only considered correct if they fall into the tumor branch of AMKL (i.e., LEUK for level 1 classifier, AML for level 2, and AMKL for level 3). It may happen that the prediction labels are correct for the level 1 and 2 classifiers but incorrect for the level 3 classifier. For the level 4 classifier, its prediction label is correct only if the prediction is one of the two AMKL subtypes (i.e., AMKL CBFA2T3-GLIS2 or AMKL HOX). The sample's prediction labels by classifiers 5 to 8 are not used. This metric treats the prediction labels of FALSE samples in different classifiers individually, so a sample may be correctly classified in one classifier but incorrectly classified in another. Since the previous metric does not consider the hierarchical relationship in tumor subtypes, we combine prediction labels at different levels and compute **combined accuracy** to evaluate the performance of the classifiers. For this metric, a sample is considered correctly predicted by a level *n* classifier only if its current label (level *n*) and previous labels (level < *n*) match a corresponding tumor branch in the reference table. For instance, if a sample has a clinical diagnosis of AMKL whose maximum level is 4, its prediction label by the level 4 classifier is considered correct only if its prediction label falls into either branch of the tumor (i.e., LEUK->AML->AMKL-> AMKL CBFA2T3-GLIS2 or LEUK->AML->AMKL-> AMKL HOX). So, the label at different levels is combined and not individually considered for each sample. Although this metric considers relationships in tumor subtypes, a sample’s labels are used repeatedly to evaluate different classifiers, making the evaluation redundant. Therefore, we define **sample-level accuracy**, which gives an overall score to the classifiers using the 887 FALSE samples’ prediction labels. Specifically, if a sample's prediction labels fall into a consistent tumor branch, it is considered a correct prediction otherwise false. For example, for an AMKL sample, the sample is considered correct only if its prediction labels up to level 4 fall into one of the two AMKL branches. The detailed prediction results on all FALSE samples can be found in SM Excel S5a.

## **Supplementary tables**

**Table S1 Statistics of primary tumors, their subtype labels, and sample sizes.** The columns show the identifiers of the 26 primary tumors, their full names, maximum labeling levels (1 is the primary category and 8 is the category with the most details on tumor characteristics), and sample sizes.

| Primary tumor | Tumor name | Max level | Sample size |
| --- | --- | --- | --- |
| T000 CNS | Central nervous system tumor | 8 | 842 |
| T001 NEBLA | Neuroblastoma | 2 | 178 |
| T002 MESODM STEMlow | Mesodermal tumor, low stemness | 5 | 353 |
| T003 MESODM STEMhigh | Mesodermal tumor, high stemness | 5 | 415 |
| T004 EWING | Ewing sarcoma | 1 | 70 |
| T005 LEUK | Leukemia | 8 | 736 |
| T006 LYMPH | Lymphoma | 3 | 94 |
| T007 THCA | Thyroid carcinoma | 4 | 514 |
| T008 THYM | Thymoma | 3 | 110 |
| T009 PCPG | Pheochromocytoma and paraganglioma | 5 | 179 |
| T010 GI | Gastrointestinal tumor | 5 | 867 |
| T011 LUAD | Lung adenocarcinoma | 5 | 491 |
| T012 SCC/BLCA | Squamous cell or bladder carcinoma | 5 | 1571 |
| T013 MELA | Melanoma | 4 | 526 |
| T014 BRCA noBAS | Breast cancer, non-basal | 4 | 903 |
| T015 BRCA BAS | Breast cancer, basal-like | 3 | 178 |
| T016 HEPAC | Hepatocellular carcinoma and cholangioma | 4 | 416 |
| T017 PAAD | Pancreatic ductal adenocarcinoma | 5 | 164 |
| T018 ACC | Adrenocortical carcinoma | 2 | 78 |
| T019 KICC | Clear cell renal carcinoma | 4 | 495 |
| T020 KIPCC | Papillary cell renal carcinoma | 4 | 276 |
| T021 KICH | Kidney chromophobe and other renal carcinoma | 2 | 60 |
| T022 UCEC/CECC | Uterine tumor | 4 | 245 |
| T023 OV | Ovarian cystadenocarcinoma | 3 | 422 |
| T024 PRAD | Prostate adenocarcinoma | 4 | 332 |
| T025 TGCT SEM | Testicular germ cell tumor, seminoma | 1 | 74 |

**Table S2 Statistics of labels at different levels.** The table shows the number of samples that are in the TRUE group (i.e., the samples that correctly clustered in the original paper) and the number of labels at each level. The last three rows are the total number of samples and labels in the TRUE and FALSE groups, respectively, and their sum. NA: not available.

| Tier level | # of labels | # of samples |
| --- | --- | --- |
| Level 1 | 26 | 10,589 |
| Level 2 | 56 | 10,316 |
| Level 3 | 114 | 9,630 |
| Level 4 | 126 | 6,899 |
| Level 5 | 59 | 2,814 |
| Level 6 | 9 | 669 |
| Level 7 | 10 | 560 |
| Level 8 | 5 | 157 |
| TRUE | 405 | 10,589 |
| FALSE | NA | 887 |
| ALL | 405 | 11,476 |

**Table S3 Samples with label issues identified from (Comitani *et al.*, 2023).** Duplicate means the same label shows up more than one times at different levels with different identifiers (e.g., T169). This is not supposed to happen because they represent different tumor subtypes. Mismatch means the child label does not match with its mother label. For instance, we replace T168 THCA BRAF with T168 THCA BRAF+RAS because it has subtypes that contains BRAF or RAS mutations. Overall, the number of labels with issues are quite small, so it should have little or no impact on the classifiers’ performance.

| Original label | Error description | Modified label |
| --- | --- | --- |
| T169 THCA RAS | Duplicate; Mismatch | T169 THCA NBNR |
| T183 THCA RAS 1 | Duplicate | T183 THCA NBNR 1 |
| T168 THCA BRAF | Mismatch | T168 THCA BRAF+RAS |
| T170 THCA BRAF 1 | Duplicate | T170 THCA RAS |
| T171 THCA BRAF 2 | Duplicate | T171 THCA 1 |
| T172 THCA BRAF 3 | Duplicate | T172 THCA 2 |
| T173 THCA BRAF 4 | Mismatch | T173 THCA BRAF |
| T174 THCA BRAF 5 | Mismatch | T174 THCA 3 |
| T395 PRAAD ETS+ERG 1 | Duplicate | T395 PRAAD ETS+ERG 2 |
| T246 LUSC SEC  T240 LUSC SEC | Duplicate | Untouched |

**Table S4 Statistics of genes and interactions in cancer hallmarks.** The columns are full names of cancer hallmarks with the index number at the front, the number of genes in a hallmark, the number of interactions identified from Reactome in a hallmark, the number of gene exclusive for a hallmark, and the number of genes that have no interactions in a hallmark.

| Hallmark name | # of Gene | # of FI | Gene  (exclusive) | Gene  (without FI) | |
| --- | --- | --- | --- | --- | --- |
| 0_Sustaining Proliferative Signal | 3,747 | 57,223 | 727 | 877 |  |
| 1_Evading Growth Suppressor | 2,597 | 37,589 | 64 | 684 |  |
| 2_Resist Cell Death | 579 | 16,378 | 163 | 91 |  |
| 3_Enabling Replicative Immortality | 188 | 7,154 | 59 | 41 |  |
| 4_Inducing Angiogenesis | 585 | 15,410 | 146 | 93 |  |
| 5_Activating Invasion and Metastasis | 1,827 | 37,206 | 909 | 352 |  |
| 6_Genome Instability and Mutation | 511 | 14,289 | 278 | 68 |  |
| 7_Tumor promoting Inflammation | 649 | 15,268 | 147 | 92 |  |
| 8_Deregulating Cellular Energetic | 242 | 6,136 | 105 | 49 |  |
| 9_Avoiding Immune Destruction | 776 | 19,541 | 371 | 106 |  |

**Table S5 The mathematical definitions of the metrics used to evaluate the classifiers.** TP is True Positive, TN is True Negative, FP is False Positive, and FN is False Negative. All derived from the confusion matrix. C is the total number of labels used to train a classifier, TP_i_ and FP_i_ are the True and False Positives of a label i, respectively.

| Metric | Math Definition |
| --- | --- |
| Accuracy | $\frac{TP+FN}{TP+TN+FP+FN}$ |
| Balanced Accuracy | $\frac{Sensitivity(\frac{TP}{TP+FN})+Specificity(\frac{TN}{TN+FP})}{2}$ |
| Macro Precision | $\frac{1}{C}\sum_{i=1}^{C} \frac{TP_{i}}{TP_{i}+FP_{i}}$ |
| Weighted Precision | $\frac{\sum_{i=1}^{C} W_{i}\frac{TP_{i}}{TP_{i}+FP_{i}}}{\sum_{i=1}^{C} W_{i}}$ |
| Macro Recall | $\frac{1}{C}\sum_{i=1}^{C} \frac{TP_{i}}{TP_{i}+FN_{i}}$ |
| Macro F1 | $\frac{1}{C}\sum_{i=1}^{C} \frac{2TP_{i}}{2TP_{i}+FP_{i}+FN_{i}}$ |
| Weighted F1 | $\frac{\sum_{i=1}^{C} W_{i}\frac{2TP_{i}}{2TP_{i}+FP_{i}+FN_{i}}}{\sum_{i=1}^{C} W_{i}}$ |
| AUROC | $\int_{0}^{1} TPR(FPR)d_{FPR}$ |
| AUPRC | $\int_{0}^{1} Precision(Recall)d_{Recall}$ |

## **Supplementary figures**

##
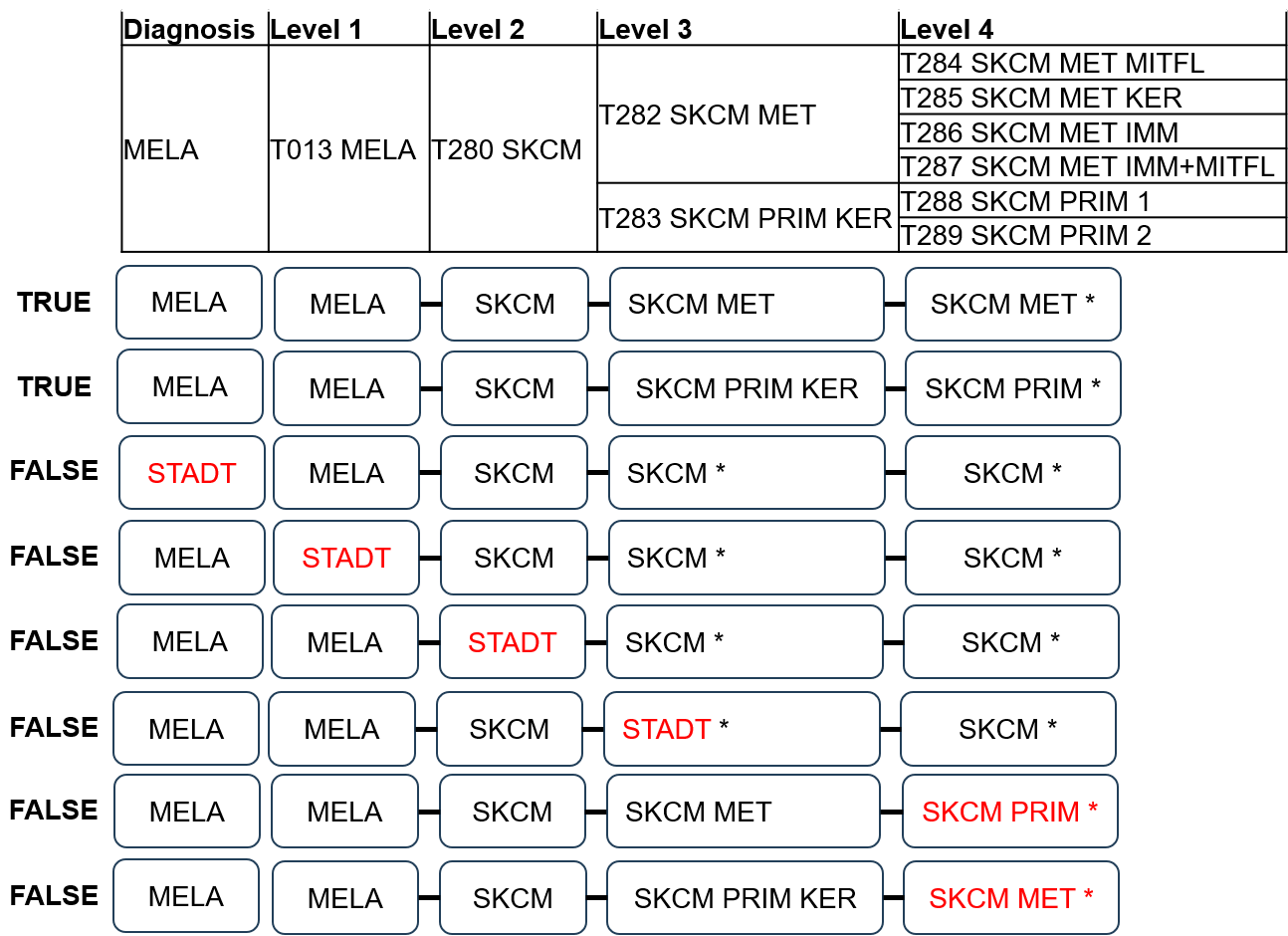


**Figure S1 An example for sample categorization.** Melanoma (MELA) labels have four levels and differentiate subtypes at levels 3 and 4 (table extracted from SM Excel S3). A melanoma sample is categorized into the TRUE group only if its diagnosis is MELA and one of its corresponding labels (rectangles) in the original study has the matching keyword for levels (e.g., MELA for level 1 and SKCM for levels 2-4). Otherwise, a mismatch in diagnosis or any of the levels will place it in the FALSE group. For example, the sample's diagnosis and level 1-3 labels do not match MELA or SKCM, or the sample's level 4 subtype does not match its level 3 subtype. The samples in the TRUE group are used for model training and those in the FALSE group are used for model validation. The asterisk indicates characters that elaborate tumor subtypes.


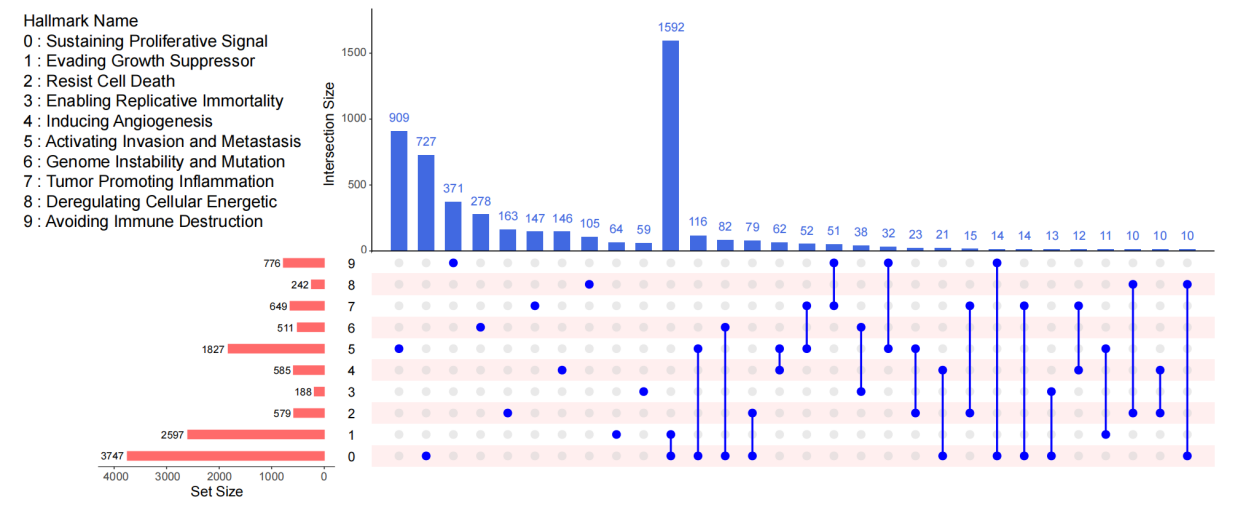


**Figure S2 Hallmark gene set analysis.** The red bars are the number of genes in specific hallmark sets. The blue bars show the number of genes in specific gene sets or overlap between two or more gene sets. The single blue dots indicate a single gene set and the connected blue dots indicate the shared gene sets.


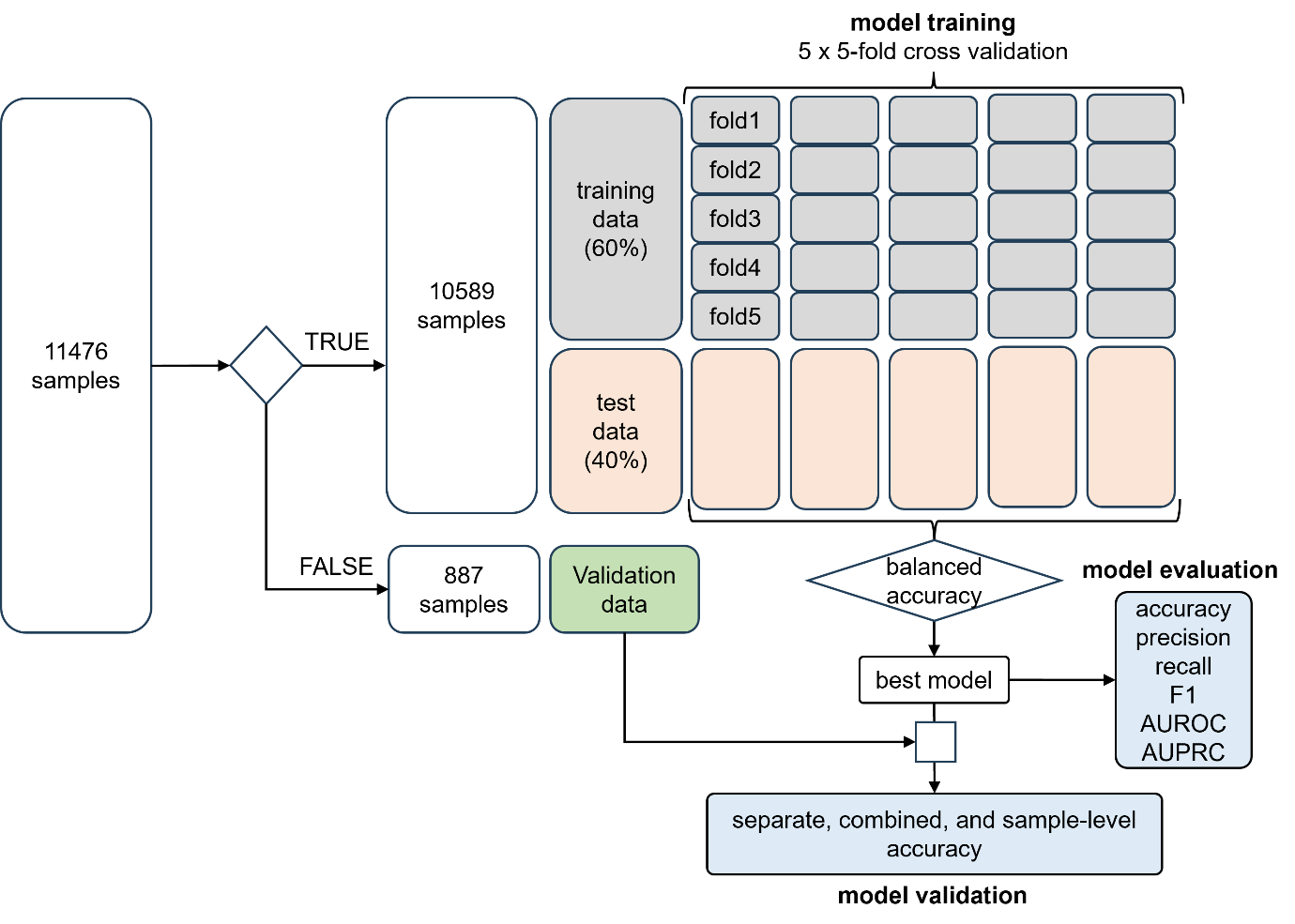


**Figure S3 The overview of data split, model training, evaluation, and validation.** All 11,476 samples are divided into TRUE and FALSE groups based on whether they are corrected labeled in the original study. This results in 10,589 samples for model training and 887 samples for model validation. Before training, the TRUE group samples are split into 60% training and 40% test data sets. During training, the 5-fold cross-validation is repeated five times. The resulting five models are evaluated using the test data, and the model with the highest balance accuracy is selected as the best model, whose performance is evaluated using standard metrics (see main text Table 1). Finally, the best model is used to predict the validation data, and the results are used to evaluate the performance of the classifiers using the three defined metrics (see Supplementary Texts for details).


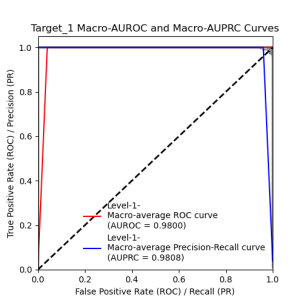

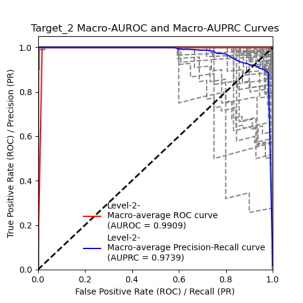

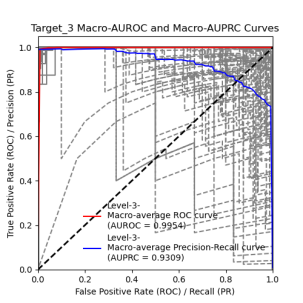

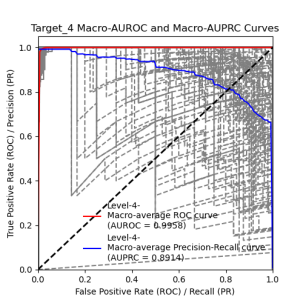

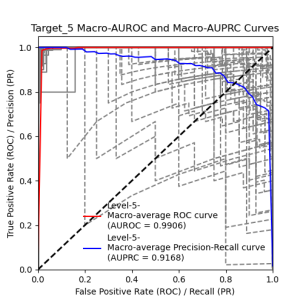

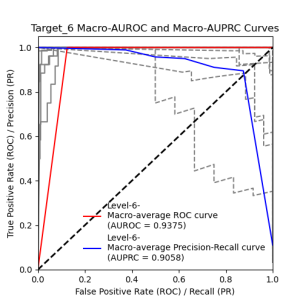

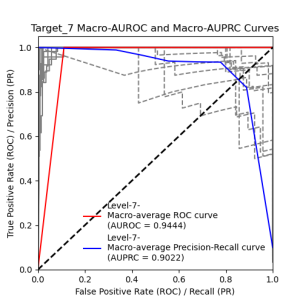

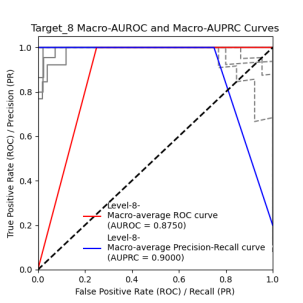


**Figure S4 The ROC and PRC plots for the eight classifiers trained with 60% training and 40% test data.** The red and blue dashed lines represent the macro AUROC and macro AUPRC, respectively. The gray solid and dashed lines represent individual ROC and PR curves for different true labels, respectively. The top plots are for classifiers for 1-3 level labels (from left to right), the middle plots are for level 4-6 classifiers (from left to right), and the bottom are for level 7 and 8 classifiers (from left to right).


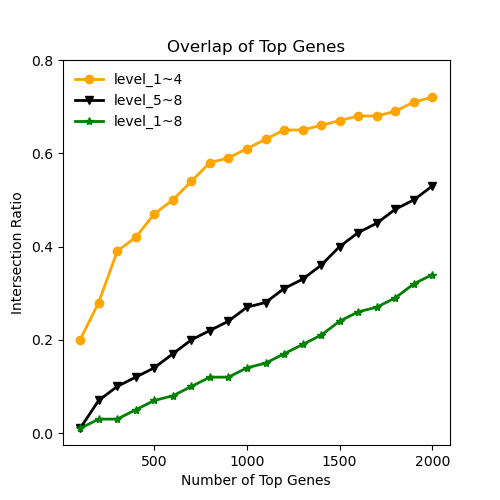


**Figure S5 The model performance with 80% training and 20% test data.** The lines indicate the overlap of genes in different classifier while increasing the number of top-ranking genes from 100 to 2000.


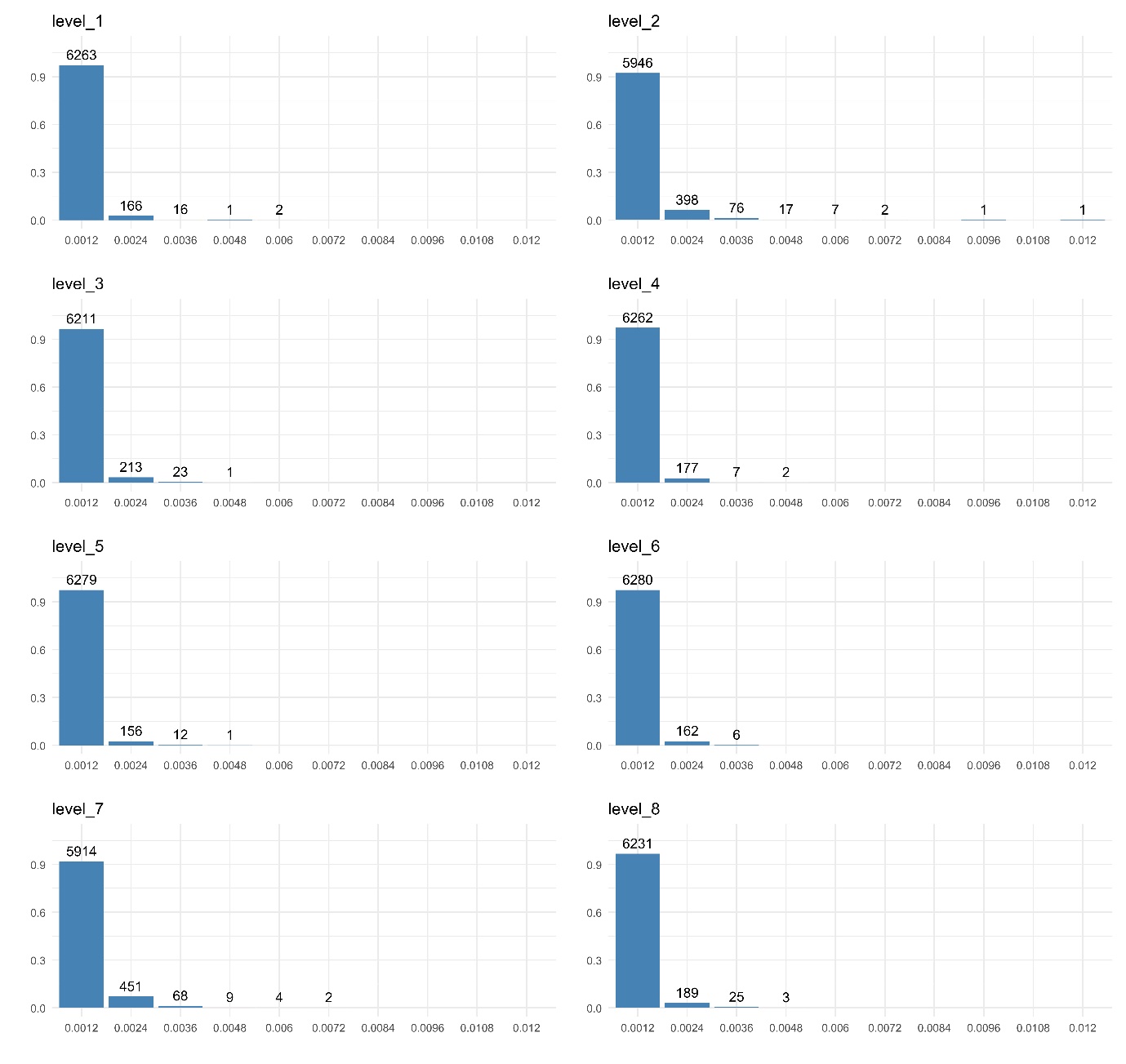


**Figure S6 The histogram of genes’ absolute, average SHAP values in each classifier.** The absolute, averaged SHAP values of most genes are similar and small in all classifiers. The classifier 2 has a wider spread towards the bigger values.


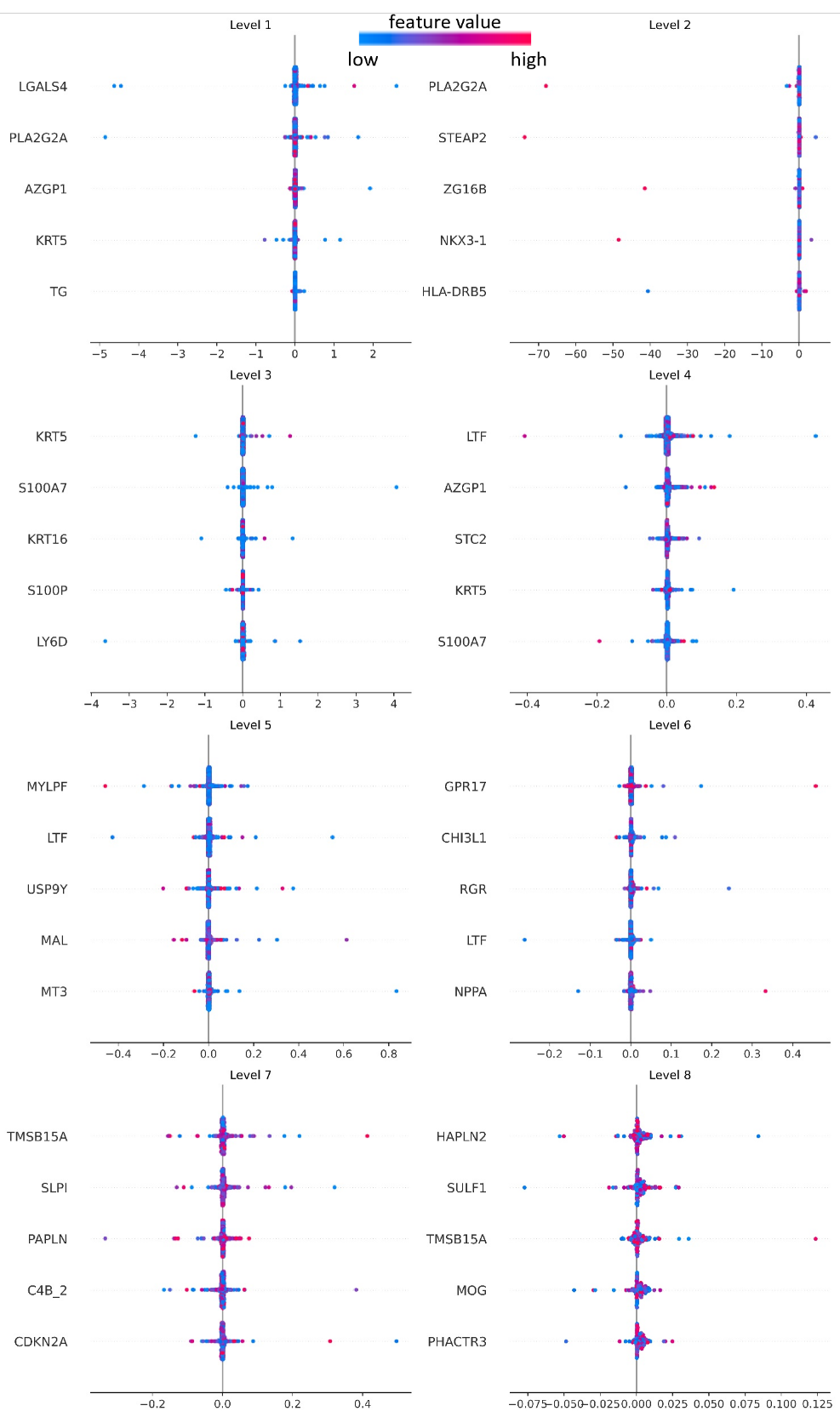


**Figure S7 SHAP values of the top 5 ranked genes in the classifiers.** Each panel shows a plot illustrating the SHAP values of the top 5 ranked genes in individual samples of a classifier. Each point represents a sample with its SHAP value on the x-axis and its feature value coded by color. Of note, the level 2 classifier's top-ranked genes have extremely high SHAP values for a few samples.


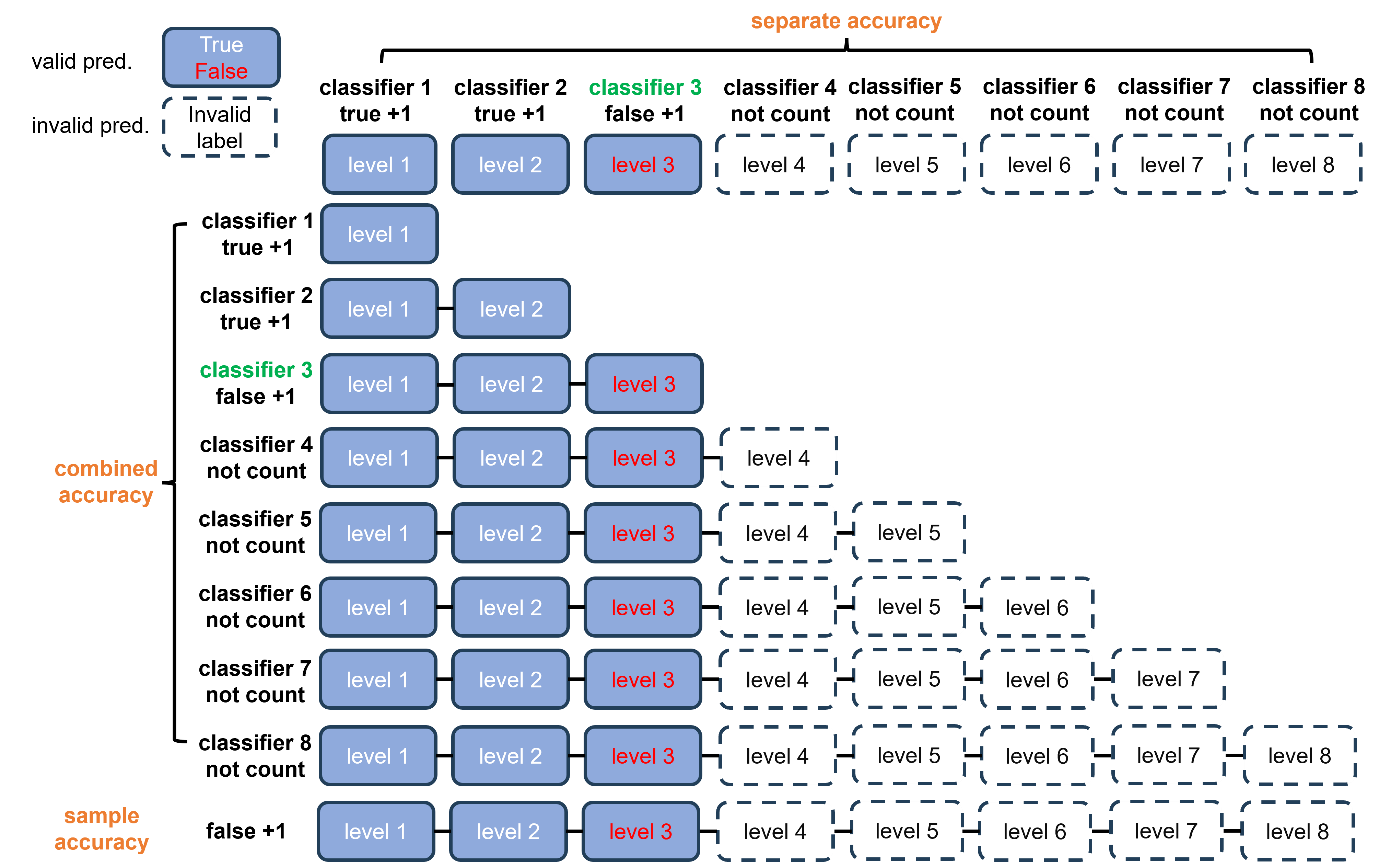


**Figure S8 The computation of the separate, combined, and sample-level accuracy using the predictive labels of the FALSE group’s 887 samples.** Here is an example for a sample whose right maximum level is 3 (highlighted in green) and determined by its original diagnosis. The sample’s labels predicted by the classifiers 1, 2, and 3 (filled rectangles with white and red fonts representing true and false predictions, respectively) are used to compute the corresponding accuracy. Other predictive labels beyond level 3 (i.e., level 4-8) are considered invalid (empty rectangles), and are therefore not used to compute the three metrics. For separated accuracy, the level 1 and 2 classifiers correctly predict its labels in the level 1 and 2 classifiers, but the level 3 classifier’s prediction is incorrect. For combined accuracy, only the predictions of the level 1 and 2 are corrected, and the level 3 classifier is wrong. For the sample-level accuracy, the sample is counted as a false prediction because its level 3 prediction is incorrect.
